# Differential effects of sodium chloride and monosodium glutamate on kidney of adult and aging mice

**DOI:** 10.1038/s41598-020-80048-z

**Published:** 2021-01-12

**Authors:** Michele Celestino, Valeria Balmaceda Valdez, Paola Brun, Ignazio Castagliuolo, Carla Mucignat-Caretta

**Affiliations:** 1 Department of Molecular Medicine, University of Padova, 35131 Padua, Italy; 2 Biostructures and Biosystems National Institute, 00136 Rome, Italy

**Keywords:** Developmental biology, Physiology

## Abstract

Monosodium Glutamate (MSG) is used as flavour enhancer, with potential beneficial effects due to its nutritional value. Given the decline in kidney functions during aging, we investigated the impact of MSG voluntary intake on the kidney of male mice, aged 6 or 18 months. For 2 months, they freely consumed water (control group), sodium chloride (0.3% NaCl) or MSG (1% MSG) in addition to standard diet. Young animals consuming sodium chloride presented signs of proteinuria, hyperfiltration, enhanced expression and excretion of Aquaporin 2 and initial degenerative reactions suggestive of fibrosis, while MSG-consuming mice were similar to controls. In old mice, aging-related effects including proteinuria and increased renal corpuscle volume were observed in all groups. At an advanced age, MSG caused no adverse effects on the kidney compared to controls, despite the presence of a sodium moiety, similar to sodium chloride. These data show that prolonged MSG intake in mice has less impact on kidney compared to sodium chloride, that already in young animals induced some effects on kidney, possibly related to hypertension.

## Introduction

Monosodium glutamate (MSG) is a water-soluble salt of glutamate, a non-essential amino acid, normally synthesized in the body and prevalent in food proteins. MSG is mostly known for its flavour enhancement properties and is commonly added to Oriental food, canned vegetables, soups and processed meat. This compound is able to elicit the same sensory molecular mechanisms of the “Umami” taste experience^1^. Actually, the human body is not able to distinguish between glutamate naturally present in food and added one, since they are exactly the same molecules^2^. Given the significance as a building block of proteins and the quantitatively high daily ingestion of glutamate as protein constituent, free MSG and/or Umami intake has relevant potential properties for health promotion, by improving palatability and hence food intake, that could be useful in some pathological or age-related states^3^. Even during healthy aging, some deficits in nutrition may lead to age-related anorexia and/or sarcopenia. Different studies show that stimulation of Umami taste promotes salivation, taste function, appetite and weight gain, helping the overall health in elderly people^4–7^. Different studies and clinical trials have failed to identify a relationship between the consumption of MSG and possible negative effects^8^. Careful investigation of scientific literature by different food safety agencies resulted in declarations that consider MSG as safe (for USA: https://www.fda.gov/food/food-additives-petitions/questions-and-answers-monosodium-glutamate-msg; for Europe: https://www.efsa.europa.eu/en/efsajournal/pub/4910, see also^9^).

In the present study, we explored the effects of dietary supplementation with MSG for 2 months on CD1 male mice, in comparison to sodium chloride (NaCl) or water, focussing on kidney morphology and function. Two different age groups were chosen to explore the effect of NaCl or MSG supplementation, on young adult or old mice, in order to observe possible deleterious effects of MSG or NaCl on elderly mice. Starting at 4 or 16 months of age, mice were fed for 2 months with maintenance diet and water ad libitum, with free access to another bottle containing either MSG (1%), NaCl (0.3%) or water as a control. Sodium chloride supplementation was administered for its known negative impact on health^10^, to control for the effect of sodium intake which takes part also with MSG. We focused on mouse kidneys histology and proteinuria, observed at two ages, as a consequence of voluntary MSG or NaCl intake.

## Results

### Albuminuria appears with NaCl supplementation without changing water intake

**Figure 1:**
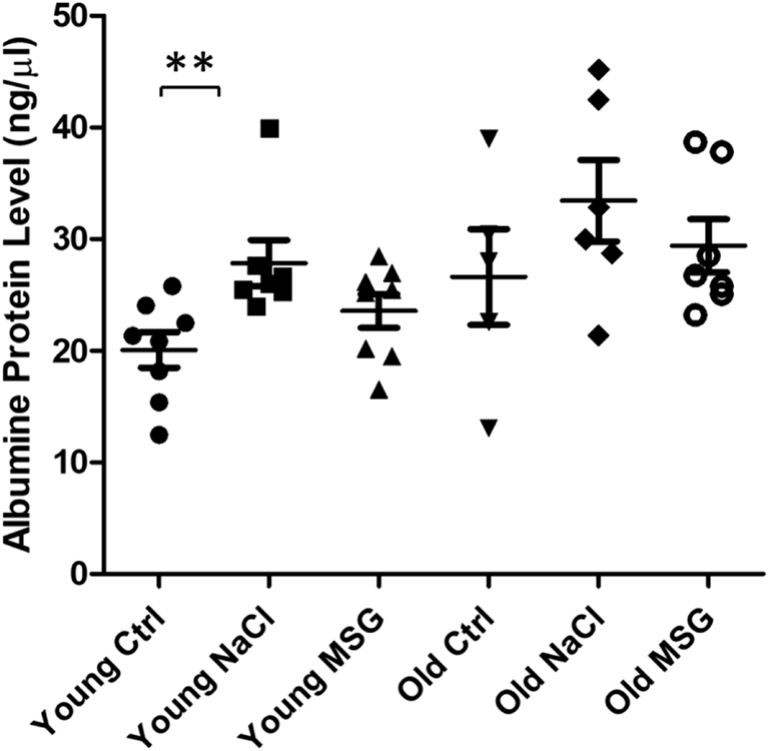
Albuminuria in male mice at the end of treatment. Albumin excretion was evaluated with silver staining. Urinary samples obtained from 6 and 18 months old mice, whose diet was supplemented for 2 months with water, sodium chloride or MSG (Ctrl, NaCl and MSG, respectively), were examined. N = 8 (young Ctrl), 7 (young NaCl), 8 (young MSG), 5 (old Ctrl), 6 (old NaCl), 7 (old MSG). Mann–Whitney test **p < 0.01.

**Figure 2:**
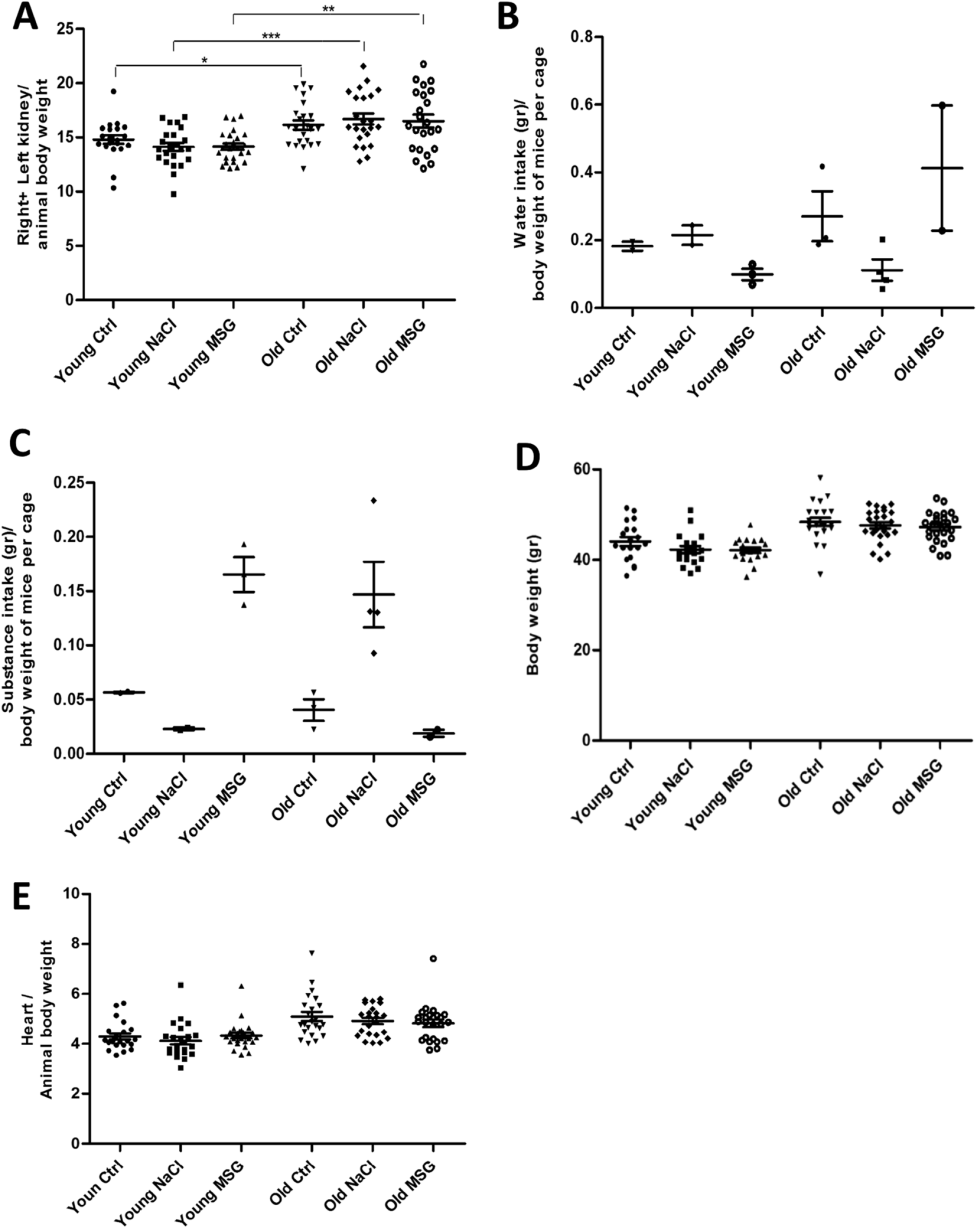
Kidney weight and water/substance intake measurements. (**A**) Ratio of total kidney (left plus right) weight to body weight in 6 months (young) or 18 months (old) old mice groups. An increase in kidney weight was associated with aging in each treatment group (n = 21 in each group. *t* test: *p < 0.05, **p < 0.01, ***p < 0.001). (**B, C**) Water (**B**) and (**C**) substance (Water, NaCl and MSG respectively) consumption normalized by total mouse weight within a cage. (**D**) Body weight at the end of treatment. (**E**) Heart weight normalized to body weight (mg/g) at the end of treatment.

Initially, albumin was quantified in urine in order to evaluate a possible kidney damage. Albumin levels measured at the end of the 2 months treatment indicated that aging, as expected, induced a slight non-significant increase in albumin excretion. However, in young NaCl group, excretion of albumin was significantly higher if compared to control animals (Fig. 1 and Supplementary Fig. 1A); a similar trend was also present when the percentage of total proteins in urine was analysed. Interestingly, MSG did not cause an increase in albumin excretion in urine (Supplementary Fig. 1B,C). No differences were detected in the kidney weight among treatments (Fig. 2A), apart from the physiological increase in old compared to young mice. Next, we evaluated water intake at the end of the supplementation period, without finding significant differences (Fig. 2B). Regarding the substance intake (i.e. water for control mice, NaCl for NaCl and MSG for MSG mice, respectively) no statistically significant differences were apparent (Fig. 2C). Also, body weight and heart weight, normalized to body weight did not differ (Fig. 2D,E).

### Glomerular volume increases upon NaCl addition

**Figure 3:**
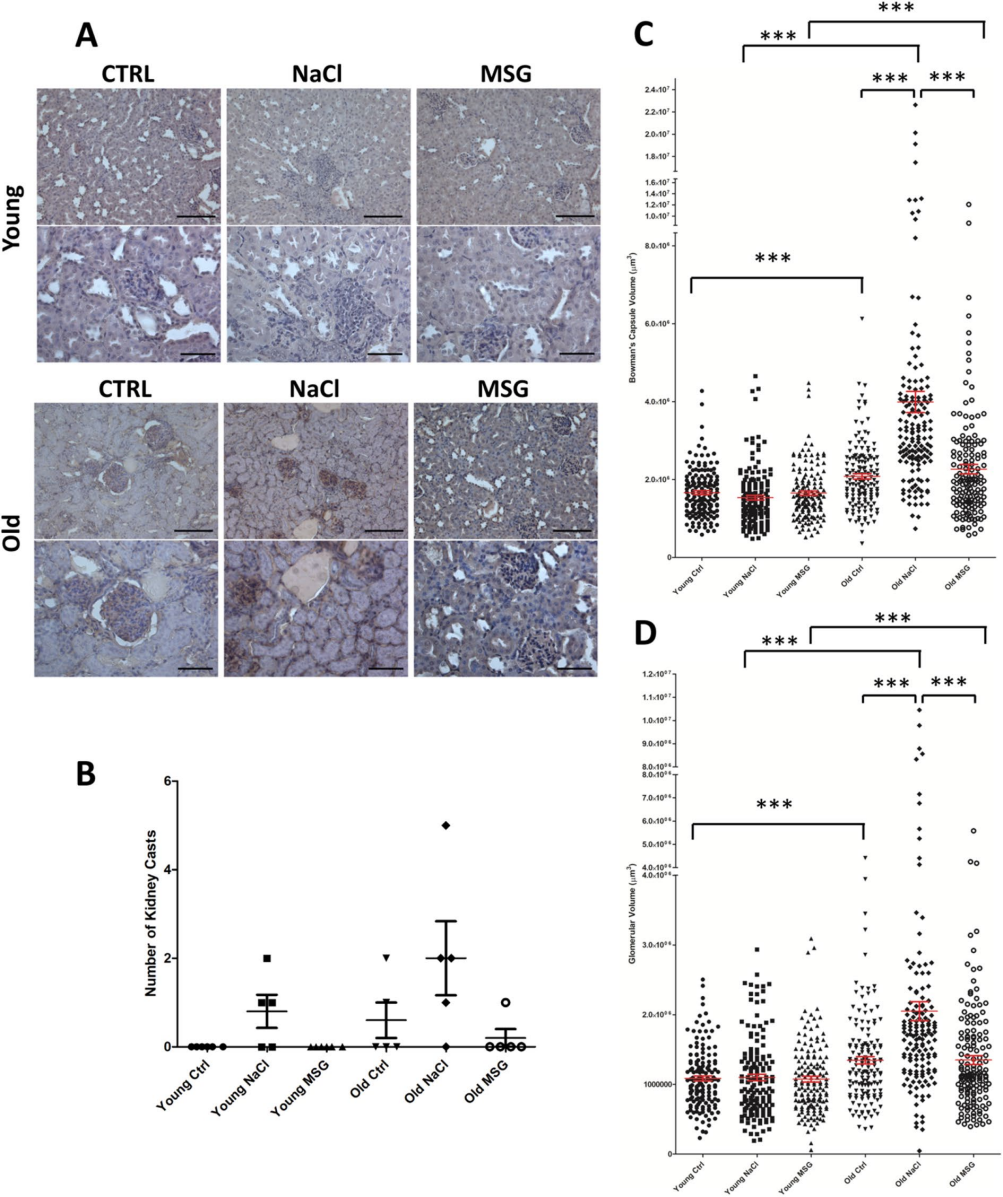
Effect of diet in association with age on renal phenotype. (**A**) Kidney PAS staining was performed on 5 male mice randomly selected within each group. Black arrow points to proteinaceous casts and black stars areas mark increased cellular proliferation, respectively. (**B**) Mean number of casts in each group (n = 5 mice/group), no casts were detected in young control and MSG groups. In old mice, NaCl versus MSG group Mann–Whitney U = 3.50, *p* = 0.056. (**C, D**) Bowman’s Capsule volume (**C**) and Glomerular volume (**D**) morphometric measures were performed in PAS-stained tissues. N = 150 glomeruli from 5 mice in each group. *t* test, *p < 0.05, **p < 0.01, ***p < 0.001.

Renal histopathology of the glomerular and tubulointerstitial areas revealed the presence of intratubular casts with different morphology and size in some groups of mice (Fig. 3A, black arrow). Casts were not present in young control and MSG groups (Fig. 3B). Moreover, an increase in cellular proliferation within the renal tissue (Fig. 3A, black star) was also observed in some animals. Morphometric measurements on PAS images^11^ showed a significant expansion of the glomerular and the Bowman’s capsule volume with aging^12^, with an additional significant increase in old NaCl group, compared to age-matched groups (Fig. 3C,D, Supplementary Fig. 2). Interestingly, NaCl-supplemented mice presented a larger glomerular volume already at young age, suggesting that sodium chloride may induce some hypertensive effects on young and old mice while MSG did not.

### Fibrosis is already apparent in young NaCl mice

**Figure 4:**
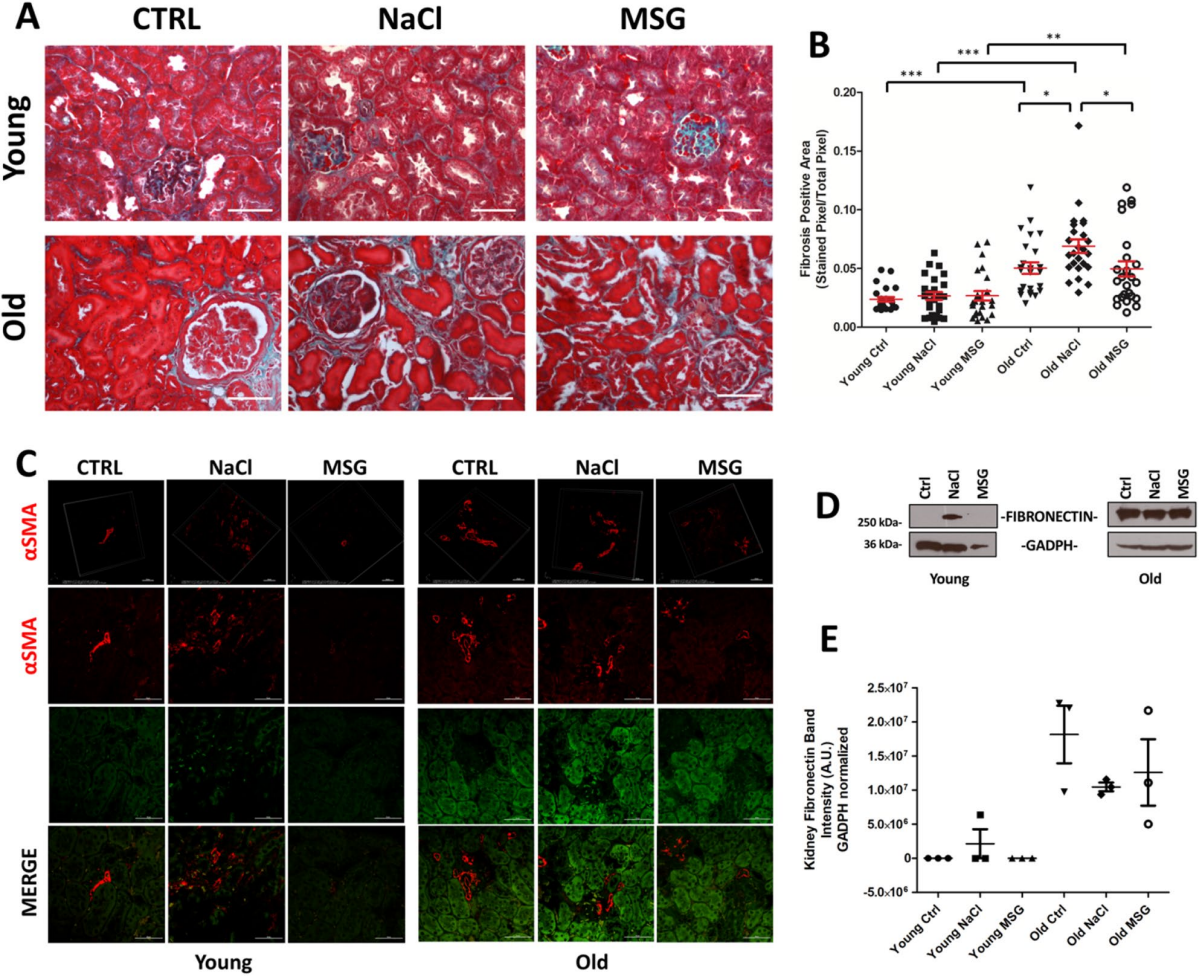
Evidence of kidney fibrosis evaluated with histology staining, αSMA labelling and fibronectin deposition. (**A**) Representative Gomori trichrome renal staining (× 40). (**B**) The dot plot analysed the fibrosis intensity in function of age and treatment (n = 25 sections from 5 mice in each group, *t* test *p < 0.05, **p < 0.01). (**C**) Immunofluorescence showing the presence of αSMA in the kidney. The green channel, unmarked, display the background and shows the morphology of the tissue. Scale bar, 50 µm. The panels in the first row represent the 3D reconstruction of the bidimensional images (scale bar 20 µm). (**D**) Fibronectin deposition in the kidney, a representative Western blot is shown. (**E**) Analysis of fibronectin deposition, as a proportion of fibronectin normalized to GAPDH (*A.U.* arbitrary units, n = 3/group). No fibronectin was detected in young control and MSG mice.

Histopathological PAS analysis of kidney sections revealed thickening of the basal membrane in some animals. Using Gomori trichrome stain, increased collagen deposition was shown in old mice, independently from the treatment (Fig. 4A). However, when the amount of fibrosis within the tissue was examined, an increase in positive area was observed in old control and NaCl groups compared to young mice, while MSG-supplemented old mice did not differ from their young counterparts (Fig. 4B).

To confirm this, we analysed the distribution of α-Smooth Muscle Actin (αSMA) to assess expansion of myofibroblast cells, that represent the major contributors of collagen I and III within the tissues^13–15^. In our samples, αSMA increased in aging mice and, surprisingly, also in NaCl young group (Fig. 4C). Also fibronectin (Fig. 4D,E), which is involved in the fibrogenesis process^16^, apparently increased within the kidney parenchyma. PAS staining revealed also some areas with increased number of cells, independently from age or treatments (Fig. 3A, black star). Together, these data suggest that fibrosis may be already apparent in young NaCl-supplemented mice and is even more evident in old controls and NaCl groups. Noticeably, this was not observed in MSG-supplemented mice at both ages.

### Aquaporin 2 expression in kidney and its urinary excretion are increased in young NaCl-supplemented mice

**Figure 5:**
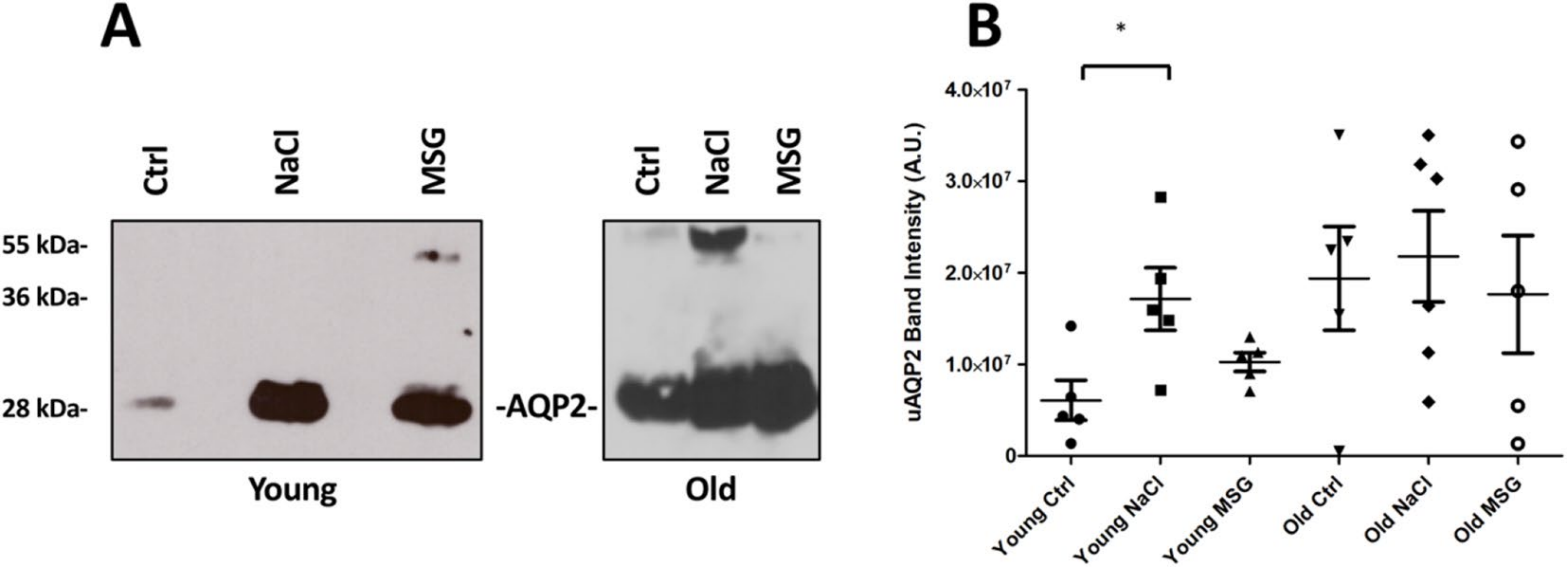
Analysis of AQP2 excretion in male mice urine. (**A**) Urine was collected from mice supplemented with water (control, Ctrl), NaCl (NaCl) or MSG (MSG) at two different end point time (young and old, 6 and 18 months respectively). A typical immunoblot with urinary samples is shown. Two bands can be seen, the top being glycosylated and the bottom non-glycosylated AQP2. (**B**) Summary of quantitative data (n = 6 in Old NaCl group, 5 in all other groups) obtained from immunoblot analysis with relative statistical analysis (*t* test, **p < 0.01).

Since both NaCl and MSG intake could increase water consumption, we evaluated the expression of the inducible Aquaporin 2 (AQP2) water channel^17^. Firstly, AQP2 was detected in urine because AQP2 excretion changes in response to antidiuretic hormone (ADH)^18^ on a salty diet^19^. As shown in Fig. 5A, Aquaporin 2 excretion was significantly higher in young NaCl group, compared to the controls (Fig. 5B). On the other hand, MSG did not impact AQP2 excretion in urine. Also, an increased release was observed with aging.

**Figure 6:**
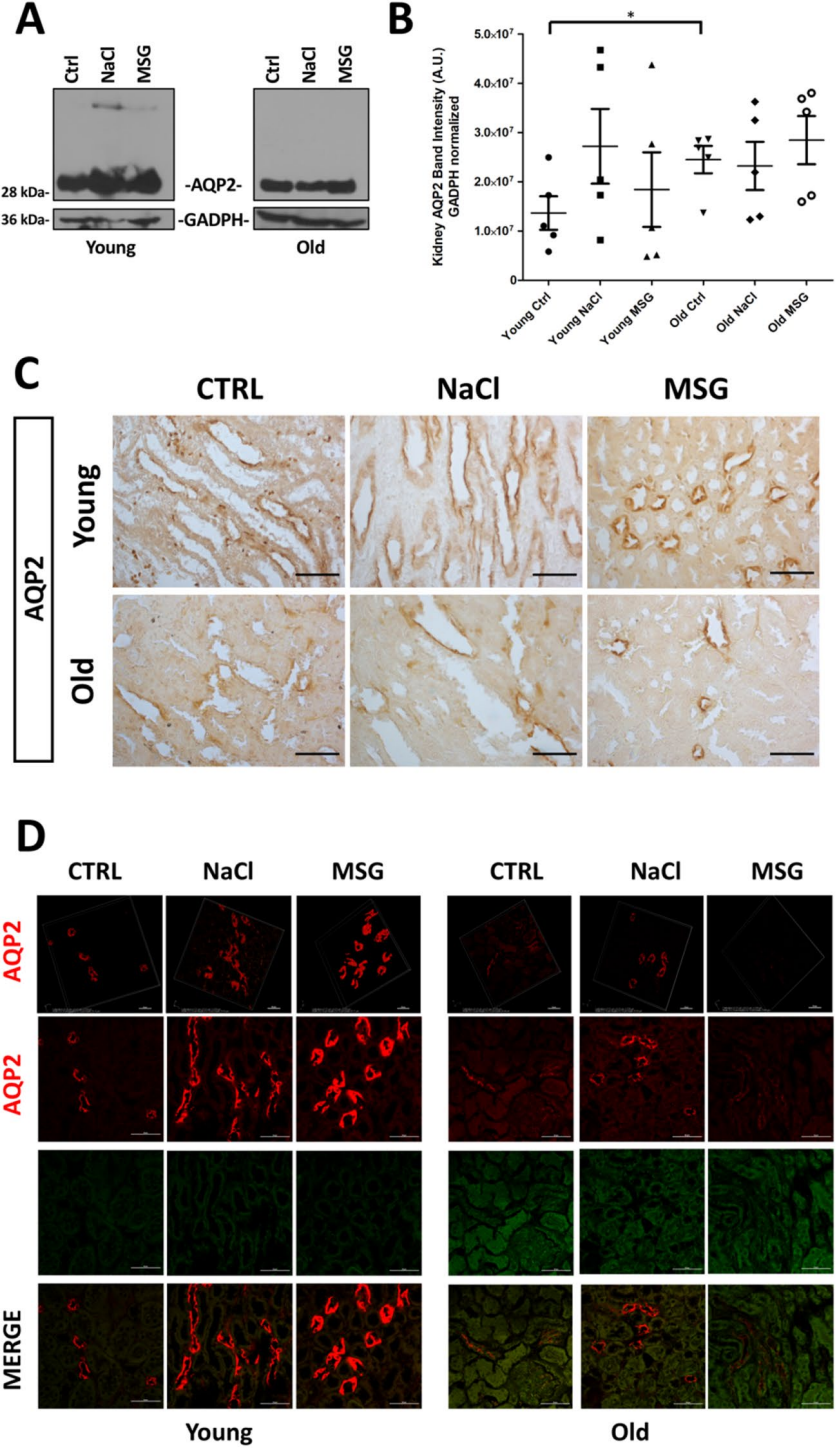
AQP2 expression and localization in kidney tissue. (**A**) Western blot analysis showing the effects of dietary supplementation and age on the expression of AQP2. (**B**) AQP2 relative intensity analysis performed on WB (*A.U.* arbitrary units, n = 5). Representative immunohistochemistry (**C**) and immunofluorescence (**D**) showing the tissue distribution of AQP2 in collecting ducts. The green channel, unmarked, displays the background and shows the morphology of the tissue. Scale bar: 50 µm. The panels in the first row represent the 3D reconstruction of AQP2 bidimensional image series (scale bar: 20 µm).

Lastly, AQP2 expression was investigated in kidney tissues (Fig. 6A). Semiquantitative Western blot analysis (Fig. 6B) showed a slightly increased expression in young NaCl group and, accordingly with the aging process^20,21^, a decrease in all old mice. Next, we evaluated also the localization of AQP2 within the kidney (Fig. 6C,D). As expected, Aquaporin 2 was present in the collecting ducts^22^ and exhibited a reduced expression in old animals (Supplementary Fig. 3A,B). In our setting, inducible water channel AQP2 decreased in old mice, independently from the treatment. Furthermore, in young mice NaCl supplementation increased both the kidney expression and urine excretion of this protein, possibly reflecting perturbation of the AQP2 balance in response to initial damage.

## Discussion

Glutamate accounts for twenty to forty percent of animal and vegetal protein weight, respectively. Within our body, one fourth of the dietary intake of amino acids belongs to the glutamate family, including glutamine, proline, histidine, arginine and ornithine besides glutamate itself^23^. Kidneys have a crucial role in regulating body homeostasis and disposal of waste substances, including amino acid metabolites. During aging their function may be impaired, leading to Chronic Kidney Disease (CKD), that is a major health issue for its impact on cardiovascular health^24,25^. Various glutamate receptors are present in this organ and may modulate its function^26^ through changes in the function of renal vasculature^27^, which is also sensitive to variations of sodium chloride dietary intake^28^. Morphological changes including proliferation of mesangial cells may be observed after high-dose MSG injections in adult rats^29^. A single injection of MSG may increase lipid peroxidation in the kidney in less than 1 hour^30^, while prolonged high-dose oral MSG administration may induce congestion and tubular swelling, besides altering antioxidant system^31^, thus priming kidney for oxidative damage. However, other laboratories reported no apparent changes in renal morphology after MSG administration in drinking water, despite increasing water intake^32^. If high-dose MSG is provided in drinking water, kidney stones have been detected in some cases^33^: however, in this work, no access to plain water was allowed to MSG rats. This may explain why we did not observe stones in our MSG mice, which freely accessed both plain water and solutions. Increase in oxidative stress markers is confirmed also by proteomic analysis: also, in this case MSG rats had access only to MSG-poised drinking water, without access to plain water, a condition which hardly can be seen in real life^34^. The effect of either MSG or NaCl added to food and water was also compared: both treatments induced hypertension, while only MSG evoked hyperfiltration followed by sodium, potassium and water reabsorption, with the involvement of NMDA receptors^35^. In this model, no increase in lipid peroxidation was observed, but fibrosis was present^36^. Despite being relevant part of our diet, several studies in the literature refer to the effects of injection of a high dose glutamate in neonatal rodents, which results in increased body weight, fat deposition, decreased motor activity, and secretion of growth hormone in the rat^37^. All these effects could culminate with induction of obesity^38^ and, ultimately, with diabetes^39,40^. Apparently, dose, duration and way of administration impact the effect of MSG on kidney.

To study the effect of MSG in physiological conditions, in the present work we compared the effects of voluntary sodium chloride or MSG dietary intake for 2 months in healthy CD1 male mice maintained with balanced diet, at two different ages, corresponding to human adult and initial third age. While blood pressure would be an interesting variable to study, it is quite hard to obtain in living mice, because tail cuff impedes free movement, while permanently implanted catheters or implanted telemetry are invasive and stressful manipulations. In our experimental setup, we aimed at studying mouse intake in a rather quiet condition, to avoid the effect of stress hormone on the kidney and on the cardiovascular system, which in turn influence kidney function. For the same reason metabolic cages were not used: in order to link data from single mice, collection of urine from a single animal should be performed while avoiding the stress of putting mice, a social species, in chronic isolation. Compared to other mouse strains, CD1 mice are a good model for metabolic studies, e.g. fatty acid trafficking, in various organs^41,42^. Initially, we detected an increase in albuminuria after NaCl supplementation, more evident in young mice, while in MSG-supplemented mice values were similar to controls. Albuminuria is a fallout of high NaCl intake in humans as well^43^ and is a major determinant of kidney fibrosis and end-stage renal failure^44^.

In our animal model supplemented with 1% MSG, the relative kidney and body weights remained unaltered during 2 months. Similar results were found in NaCl-supplemented mice, in which kidneys/body weight ratio was unaffected. Prolonged high sodium chloride diet on rats did not alter the kidney/body weight ratio used as an index of renal hypertrophy^45^ whereas it may cause hyperphagia in mice and humans without changes in weight, due to a hypercatabolic state associated to loss in the abdominal fat^46^. The mice groups that had free access to 0.3% NaCl solution showed similar liquid intake to the control animals. Under the present conditions, which include voluntary ingestion of the substance, at a lower dosage compared to other studies and in the presence also of plain water, no apparent changes were detected in kidney morphology of MSG-supplemented mice. NaCl-supplemented mice occasionally presented casts already at young age, and in old individuals they were more numerous. Actually, casts may be present in aging kidneys per se. Also, in rats and humans NaCl may induce alteration in the kidney architecture including casts and fibrosis^47^.

In the present study, we did not detect apparent changes in kidney morphology in MSG mice upon free access to MSG. In line with our results, other laboratories reported no alteration in renal morphology after MSG administration in drinking water^48^. Also, an extensive re-evaluation of glutamate intake effects on the kidney revealed only an increase in organ weight at high dosages, without any histopathological finding^9^. In our experimental setting, with free access to water, we did not observe the presence of stones in the kidney tissues analyzed. It must be emphasized that feeding only with MSG-poised drinking water, without access to plain water, is a condition which hardly can be seen in real life. Furthermore, our old NaCl group also showed alterations in morphometric analysis of the renal corpuscle, with a larger Bowman capsule and glomerular volume, that is often linked to hyperfiltration^49^. The impact of NaCl on kidney structure is both direct and indirect, through a general increase in systemic blood pressure^50^. Moreover, aging per se may induce glomerulosclerosis and interstitial fibrosis, that is indirectly supported by the increase of Ki67 marker^51^. Noticeably, the CD1 mice strain is particularly prone to renal fibrosis^52^. Accordingly, our old mice showed fibrosis compared to young, in particular the NaCl group. Noteworthy, MSG-supplemented old mice did not differ from young, which were similar to young controls.

One of the most plastic features of kidney is its ability to reabsorb water according to body needs. Aquaporins are post-transcriptionally downregulated in aging rat kidneys^53^, accounting for a decreased ability of aging kidneys to concentrate urine. AQP2 expression in the kidney is upregulated by water deprivation^53,54^ and also by protein load^55,56^, but neither in young nor in old MSG groups we detected an increase in this protein, if compared to age-matched controls. AQP2 urinary (uAQP2) excretion reflects short-term changes in vasopressin release, being both suppressed by water load and increased by sodium chloride administration, hence uAQP2 represents a marker for collecting duct responses to vasopressin^57^. Surprisingly, we did not detect significant changes, except for an increase in urinary excretion of AQP2 in NaCl young group, compared to controls. Furthermore, we did not observe any significant variation in MSG-supplemented mice. Lastly, sodium chloride ingestion implies that both sodium and chloride ions may act inside the body: indeed, hyperchloremia is associated to renal vasoconstriction that decreases the glomerular filtration rate, due to the autoregulatory tubule-glomerular feedback control mechanism^58^. Moreover, chloride is a modulator of transtubular transport via the sodium chloride cotransporter, functionally coupled to potassium flux^59^. Extracellular chloride may also activate a molecular switch on collagen IV which promotes scaffolding outside the cells, thus impairing kidney tissue architecture and ultimately its function^60^. Chloride channels in vascular smooth muscle cells, either activated by volume like ClC-3 or by calcium like TMEM16A, are involved in hypertension genesis^61–63^, like the electroneutral sodium–potassium-chloride cotransporters, that sense intracellular chloride in vascular smooth muscle cells and neurons (NKCC1) and in the renal tubular fluid (NKCC2)^64^. Notably, chloride gradient is the main driving force for NKCC2 activation in the thick ascending limb, thus affecting ion trafficking across the epithelial membrane^65^. Also, deregulation of claudins affect paracellular Cl– transport causing hypertension^66^. Thus, the deleterious effect detected in NaCl supplemented mice are possibly linked to both sodium and chloride ions.

## Conclusion

The increase in glomerular volume, in fibrosis and in water channels point to a possible enhancement in arterial blood pressure in mice whose diet was supplemented with sodium chloride. These changes were not apparent in mice supplemented with monosodium glutamate. Given that the intake of sodium was similar in both groups, it is tentative to speculate on the deleterious role of chlorine ions ingestion. Actually, signs of damage were apparent in young animals, indicating that they were already prone to the negative effects of NaCl. Mice exposed to MSG in our more ecological setting did not face these problems, confirming a safe use of dietary MSG in both young and old age.

## Methods

### Animal experiments

The experiments conformed to the European law on animal experiments (2010/63/EU) and were authorized by University of Padova Ethical Committee (OPBA) and the Italian Ministry of Health (permission number 102/2016PR). Male CD1 mice, 4 (Young) or 16 (Old) months old, were kept under controlled environmental conditions (temperature: 23 °C, humidity: 60%, lights on: 6:00–18:00). During the experiment, animals had free access to diet and tap water; an additional bottle was added to each cage, containing either water (Control), 0.3% w/w sodium chloride in water (NaCl, Sigma-Aldrich Milan, Italy) or 1% w/w monosodium glutamate in water (MSG, Ajinomoto Co.) for 2 months. Concentration of MSG solution was chosen to mimic human daily ingestion^67^ assuming a regular drinking activity, and mice preference tests^68^, while NaCl concentration was chosen to be equimolar in sodium to MSG solution and attractive to mice. During the last 2 weeks of treatment, food and water consumption was determined. At the end of treatment, mice were anesthetized with halothane and sacrificed by cervical dislocation. The right kidney was removed, weighted and fixed in 4% formaldehyde, dehydrated, and embedded in paraffin. The left kidney and the heart were weighted and immediately frozen in liquid nitrogen for proteins extraction.

### Urine samples and tissue preparation

All urine samples were collected at the end of the 2-month treatment, leaving mice alone for 1 h in a plastic cage without bedding. Urine was collected immediately after release, centrifuged for 10 min at 13,000 rpm and the supernatants aliquoted and stored at – 80 °C for subsequent analysis. Frozen kidney tissue was grossly minced and resuspended in a ratio 1:5 of RIPA buffer (Sigma-Aldrich Milan, Italy) in presence of protease inhibitor cocktail (Sigma-Aldrich Milan, Italy) and dissociated in a tissue glass Teflon Dounce homogenizer. The mush was then centrifuged for 1 h at 8500 rpm at 4 °C. The supernatant was collected and quantified for the proteins content with BCA protein assay kit (Pierce, Thermo Fisher Monza, Italy).

### Histology

The right kidney was used for histological analyses. The kidneys embedded in paraffin were sectioned (5 μm) and stained with periodic acid-Schiff (PAS) for overall injury or with trichrome Gomori staining for fibrosis. The images were captured using a Leica microscope (20 × and 40 × objectives). Images were acquired with the resident software at 2088 × 1550 pixels with a colour digital camera, using a blind method. For PAS staining, images were subjected to morphometric analysis (see below) and to the count of hyaline casts within the tissue. Interstitial fibrosis was quantified in Gomori stained sections. Briefly, 5 random 40 × fields of 5 mice/group were acquired with the same parameters. For each image, green channel was extracted, and the positive green pixel were counted with Adobe Photoshop. The values obtained were divided to the total pixel area, scatter plotted and subjected to statistical analysis.

### Renal corpuscle morphometric analysis

Kidney PAS staining was used according to Sasaki^11^ for measuring the Bowman’s capsule area (BA, area of the inner side of the glomerular parietal epithelial cell layers) and the glomerular area (GA, area of the outer capillary loops of the tuft) using ImageJ software^69^ (W. S. Rasband, NIH, Bethesda, MD [http://rsb.info.nih.gov/ij/]). Using the measured BA and GA, the Bowman capsule volume (BVol) and the glomerular capillary volume (GVol) were calculated according to Sasaki^11^, briefly: BVol = (BA)^3/2^ × b/d × (f)^−3^, GVol = (GA)^3/2^ × b/d × (f)^−3^, with the following values: b(dimensionless shape coefficient) = 1.38 for spheres, d (size distribution coefficient for adjusting variations in glomerular size) = 1.01, f (correction factor for paraffin shrinkage) = 0.85^11^. Thirty random 40 × fields were acquired for every kidney slice of each of different groups, further divided from the cortex to the medullary zone in three areas: upper, middle and lower. For each area, 10 glomeruli images (40 × objective) were acquired and measured.

### Western blot

Twenty-five µg of kidney proteins or 10 µl of urine were resuspended in the correct amount of Laemmli Buffer (65.8 mM Tris–HCl pH 6.8, 26.3% w/v glycerol, 2.1% SDS, 0.01% bromophenol blue and 355 mM 2-mercaptoethanol) and run on a 10% polyacrylamide gel. For urinary albumin analysis, the gel was silver stained (methods: see below). Otherwise, proteins were blotted on nitrocellulose membrane (Bio-Rad), blocked for 1 h with 5% milk in PBS Tween 0.05% and incubated overnight with primary antibodies against: Aquaporin-2 (AQP2, made in mouse, Santa Cruz Biotechnology, DBA Segrate, Italy, 1:1000 in 5% milk in PBS Tween 0.05%), GAPDH (made in rabbit, Sigma-Aldrich Milan, Italy, 1:1000 in 5% milk in PBS Tween 0.05% ), or fibronectin (made in mouse, Sigma-Aldrich Milan, Italy, 1:1000 in 5% milk in PBS Tween 0.05%). The following day the membrane was washed with PBS Tween 0.05%, incubated for 2 h with the appropriate horseradish peroxidase (HRP)-conjugated secondary antibody (mouse or rabbit, Sigma, Milan, Italy, 1:1000 in 5% milk in PBS Tween 0.05%) and revealed through chemiluminescence (Luminata Classico Western HRP Substrate, Millipore, Sigma-Aldrich Milan, Italy). GAPDH antibody staining was made after membrane stripping. Briefly, after developing, membrane was washed with PBS, incubated in gentle agitation with roughly 50 °C stripping solution (0.2 M glycine, 1% SDS, 10% Tween, pH2.2) for 15 min, followed by washing with PBS and PBS Tween 0.05%. The membrane was then incubated for 1 h with 5% milk in PBS Tween 0.05%, re-probed overnight with GAPDH antibody and developed as above. The pixel intensities of the visualized bands were determined by using Image J software. The tissue expression of AQP2 and fibronectin were obtained by normalizing the pixel intensity with the one of GAPDH, taking into consideration the appropriate controls.

### Urinary albumin silver staining

Urine was loaded in SDS-PAGE, the gel was fixed in 40% ethanol, 10% acetic acid and 50% water overnight, then washed two times for 3 min in water and placed in 0.02% sodium thiosulfate solution for 30 min. After washing the gel with water three times for 30 s, gel was incubated for minimum 1 h with 0.4% silver nitrate and 0.1% formalin at 4 °C. Following two washes with water, silver staining was developed with 3% sodium carbonate and 0.05% formalin for a time depending on the desired intensity of the staining (5–10 min). The gel was then washed in water, blocked with 5% acetic acid for 5 min and stored at 4 °C in 1% acetic acid, scanned, and quantified with ImageJ software. The intensity of albumin bands was compared to the one of a fixed quantity of standard albumin loaded in the same gel.

### Immunohistochemistry

Serial 5 µm-thick paraffin sections were cut, collected on polylysine-coated slides and dried overnight at 37 °C, deparaffinized and rehydrated, quenched for 10 min in 0.3% hydrogen peroxide and blocked with 1% normal bovine serum albumin (BSA) in PBS for 30 min. and the primary Aquaporin-2 antibody (1:100 in 1% BSA-PBS) incubated overnight at 4 °C. After PBS wash, sections were incubated with anti-mouse horseradish peroxidase (HRP)-conjugated secondary antibody (Sigma, Milan, Italy) 1:100, for 1 h at room temperature and visualized with 0.05% 3,3-diaminobenzidine tetrahydrochloride (Sigma-Aldrich, Milan, Italy) added with 0.015% hydrogen peroxide for 5–10 min. Sections were then dehydrated, coverslipped with Eukitt (Sigma-Aldrich, Milan, Italy) and observed with a Leica microscope (40 × objective). Images were acquired as above (see Histology) with the same settings within each experiment.

### Immunofluorescence

After re-hydration, sections obtained as above were treated for antigen retrieval (10 mM citrate buffer, pH 6.0, 95–100 °C for 10 min). After PBS washing, sections were blocked with 1% normal bovine serum albumin (BSA) in PBS for 30 min. Subsequently, α-Smooth Muscle Actin-Cy3 conjugated antibody (αSMA, Sigma-Aldrich, Milan, Italy) diluted 1:150 in 1% BSA-PBS or the primary AQP2 antibody diluted 1:100 in 1% BSA-PBS was applied overnight at 4 °C. The following day, the samples were washed for 5 min in PBS, and AQP2 slides were incubated for 2 h with Alexa Fluor 594 anti-mouse secondary antibody (Sigma, Milan, Italy, 1:200 in 1% BSA-PBS). After PBS washes, sections were coverslipped with a solution of Glycerol-PBS (70% v/v) and observed with a confocal microscope (Nikon A1), maintaining constant laser acquisition settings. Three-dimensional analysis was carried on with the Nikon A1 NIS Elements Image software (v. 4.13). For αSMA images, highly stained vessels were not considered.

### Statistical analysis

All the values are presented as the mean ± standard error of the mean (SEM). For normally distributed samples, determined with Shapiro–Wilks test, two-tailed *t* Student test analysis was run between two groups, as indicated in figures. For low represented samples, Mann–Whitney non-parametric tests were performed (Prism 5; Graphpad Software, San Diego, CA). For categorical variables, chi-square test was used. *p* values less than 0.05 were considered significant and were expressed as follows: *p < 0.05; **p < 0.01; ***p < 0.001.

## Supplementary Information

Supplementary Information.
