# Supplementary material for: Differential effects of sodium chloride and monosodium glutamate on kidney of adult and aging mice
Source: Sci Rep. 2021 Jan 12;11:481. doi: 10.1038/s41598-020-80048-z (PMC7804302; doi:10.1038/s41598-020-80048-z)
Supplement: Supplementary file 1 — Supplementary Information. [file 41598_2020_80048_MOESM1_ESM.pdf]

SUPPLEMENTAL MATERIAL

DIFFERENTIAL EFFECTS OF SODIUM CHLORIDE AND MONOSODIUM GLUTAMATE ON KIDNEY OF ADULT AND AGING MICE

Michele Celestino[1], Valeria Balmaceda Valdez[1], Paola Brun[1],

Ignazio Castagliuolo[1], Carla Mucignat-Caretta[1, 2]*

[1] Department of Molecular Medicine, University of Padova, Padova 35131, Italy

[2] Biostructures and Biosystems National Institute, Rome 00136, Italy

Correspondence: carla.mucignat@unipd.it

- Supplementary Methods
- Supplementary Fig. 1 **Male mice urine proteins analysis**
- Supplementary Fig. 2 **Morphometric measure and percentage of glomeruli with no apparent Bowman's space**
- Supplementary Fig. 3 **Kidney AQP2 histochemistry quantifications**
- Supplementary Fig. 4 –Original unmodified blot
- Supplementary Fig. 5 - Original unmodified blot
- Supplementary Fig. 6 – Original unmodified blot
- Supplementary Fig. 7 – Original unmodified gels

SUPPLEMENTARY INFORMATION

METHODS

Urine quantification: Urine was sampled as described in Methods, “Urine samples and tissue preparation”. Briefly, two microliters of urine were used to measure the total protein excretion by BCA assay (Thermo Fischer, Milan, Italy).

Kidney AQP2 quantification: The images obtained from immunohistochemistry were then analysed for the AQP2 chromogen intensity staining with a method previously described [1]. Briefly, sample images were opened with the open source Fiji software[2] (ImageJ) http://fiji.sc/Fiji. A circle, with constant dimensions, was drawn around the area of interest (chromogen positive or background area) and the median value returned. Next, the “reciprocal intensity” of the stained area were calculated subtracting the stained region of interest (ROI) value from the maximum intensity value of an RGB image analysed (255), that is directly proportional to the amount of chromogen present. The background was calculated with the same procedure and subtracted to the reciprocal intensity value obtained from AQP2. For additional information see also: https://www.ihcworld.com/_books/Nguyen_Protocol_Reciprocal%20Intensity%20in%20Fiji.pdf

Data were scatter-plotted and analysed statistically (t-test).

For AQP2 immunofluorescence samples, image in RGB were converted in grayscale, an outline was drawn around each cell (region of interest, ROI), area and mean fluorescence were measured, along with several adjacent background readings. The total corrected cellular fluorescence (CTCF) = integrated density – (area of selected cell × mean fluorescence of background readings) (here referred for simplicity as Intensity in the interested graph), was calculated[3].

REFERENCES

1. Nguyen, D.H., Zhou, T., Shu, J., & Mao, J.H. Quantifying chromogen intensity in immunohistochemistry via reciprocal intensity. *Cancer InCytes* 2(1):e (2013).

2. Schindelin, J. et al. Fiji: an open-source platform for biological-image analysis. *Nature methods* **9**, 676-682, https://doi.org/10.1038/nmeth.2019 (2012).

3. McCloy, R.A. et al. Partial inhibition of Cdk1 in G 2 phase overrides the SAC and decouples mitotic events. *Cell Cycle* **13**, 1400-1412, https://doi.org/10.4161/cc.28401 (2014).

SUPPLEMENTARY FIGURES

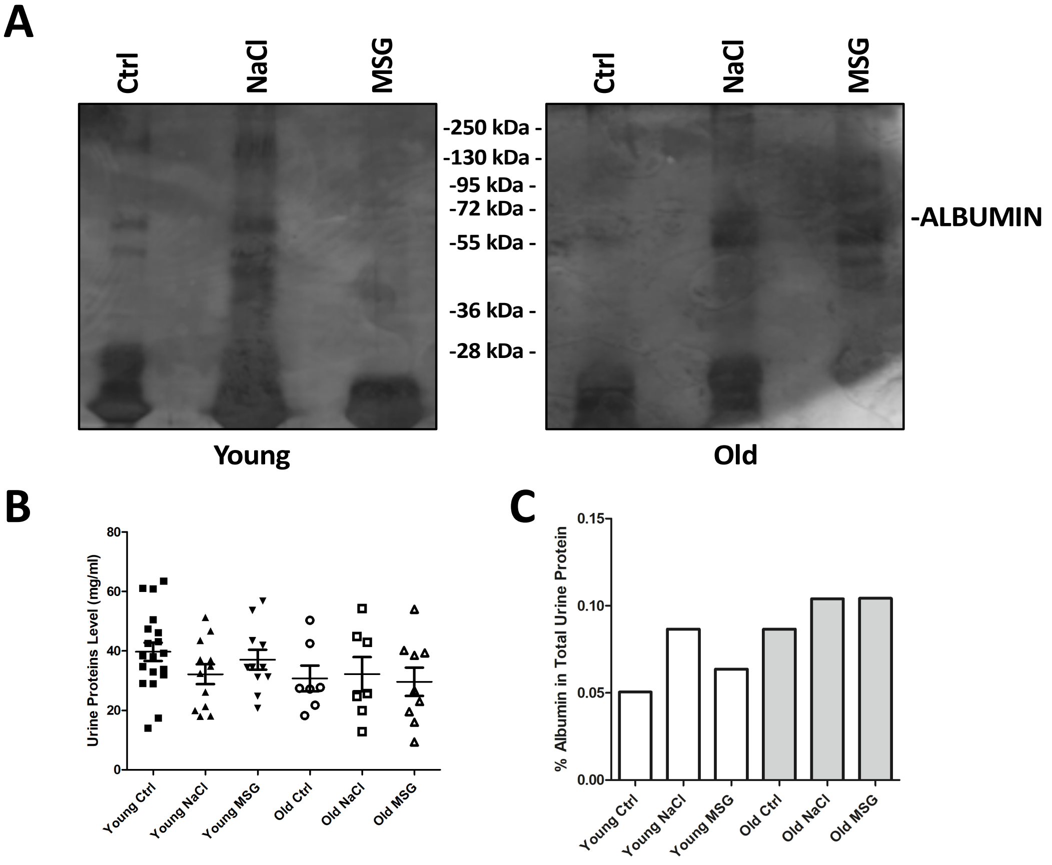

Supplementary Figure 1. Male mice urine proteins analysis. A. Silver stained SDS-PAGE gel showing protein bands visualized in 10 microliters of male mice urine. **B.** Two microliter of randomly chosen urinary samples were quantified with BCA assay for proteins amount, N = 19 (Young Ctrl), 12 (Young NaCl), 11 (Young MSG), 7 (Old Ctrl and Old NaCl), 9 (Old MSG). **C.** Percentage of albumin (see data Figure 1) referred to total urine proteins measured with BCA assay.

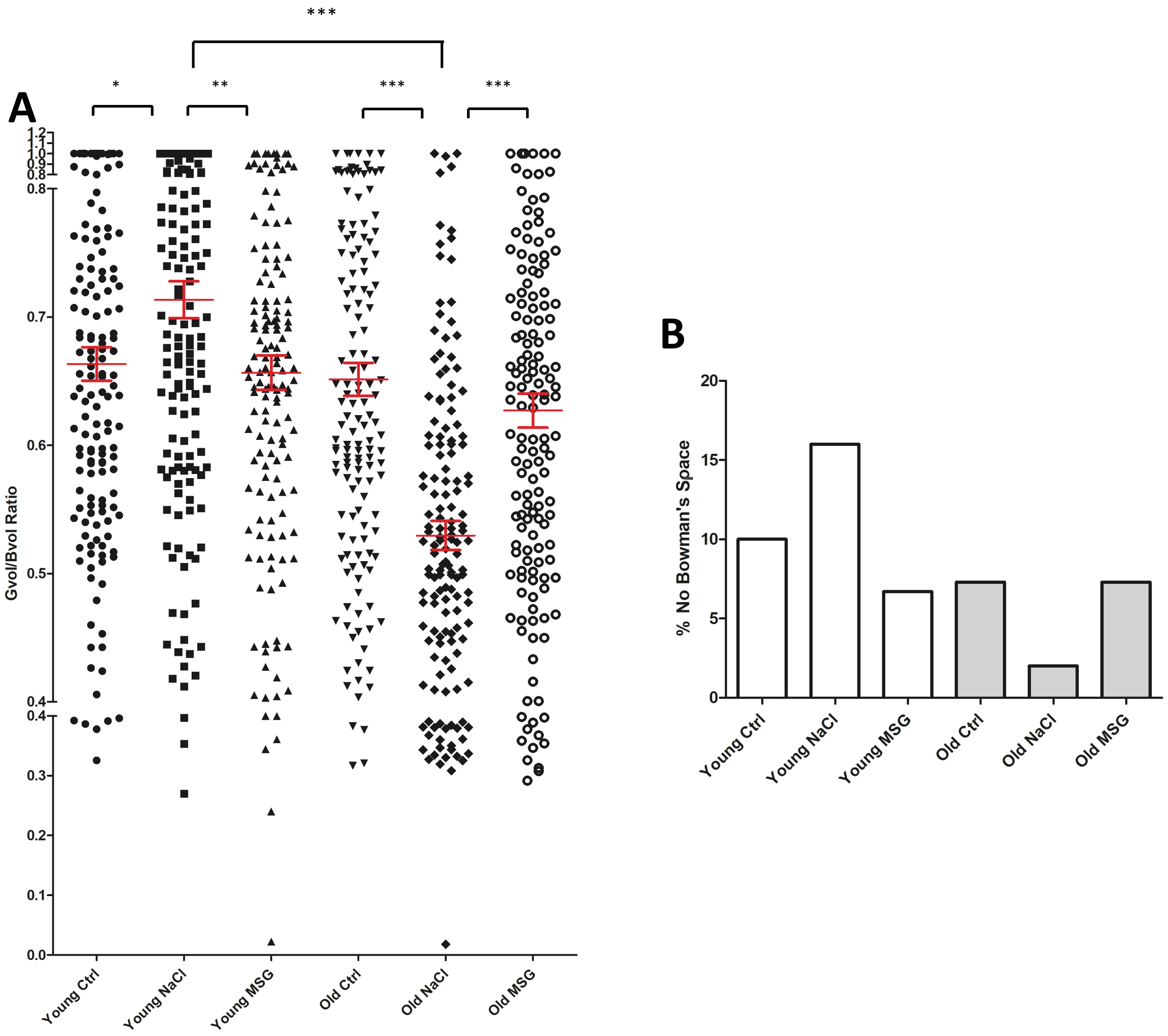

Supplementary Figure 2. Morphometric measure and percentage of glomeruli with no apparent Bowman's space. A. Elaboration of data performed on PAS staining (see data Figure 3). The scatter plot displays the ratio between glomerular and Bowman's capsule volume. **B.** The PAS images were analysed for the absence of Bowman's Capsule Space, with a different distribution between young and old mice, chi square=6.275, $p<0.05$. Data were plotted as percentage. N= 5 mice, 30 glomeruli each mouse. *$p<0.05$, ** $p>0.01$, *** $p>0.001$

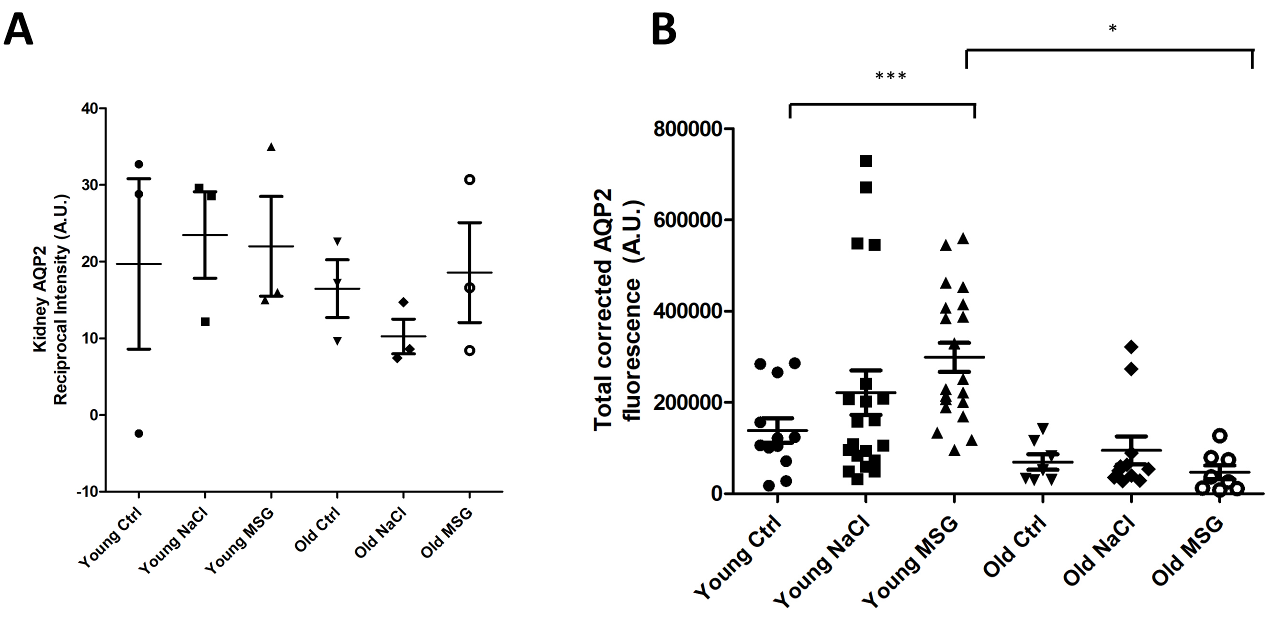

Supplementary Figure 3. Kidney AQP2 histochemistry quantifications. Immunohistochemistry and immunofluorescence images present in Figure 7 (**A** and **B** respectively) were subjected to AQP2 intensity quantification (see Supplementary Methods). T-test, *p<0.05, *** p>0.001.

SUPPLEMENTARY FIGURE 4

Original unmodified blot for Figure 4D (see main text).

Ctrl 6 months
NaCl 6 months
MSG 6 months

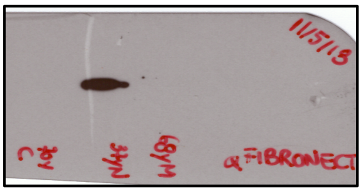

-FIBRONECTIN-

Ctrl 18 months
NaCl 18 months
MSG 18 months

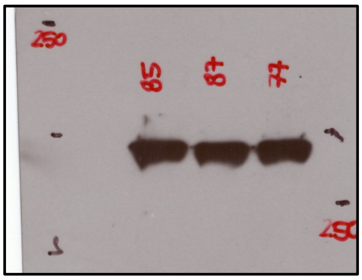

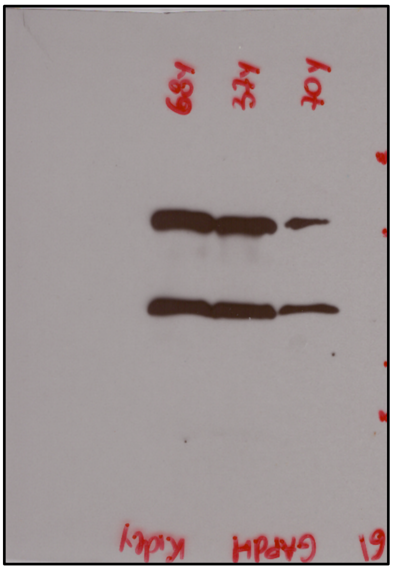

-GADPH-

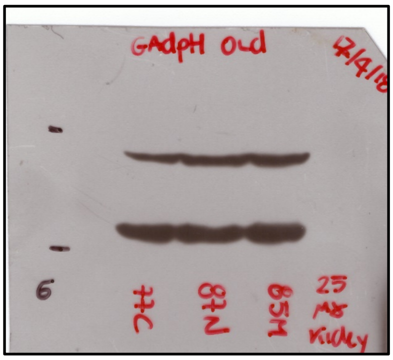

15/02/18, s2 young

21-22/02/18, s1 old

SUPPLEMENTARY FIGURE 5

Original unmodified blot for Figure 5A, for AQP2 (see main text).

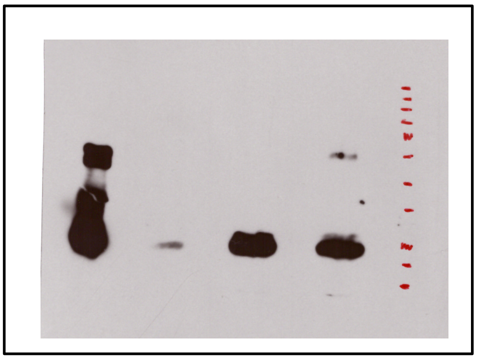

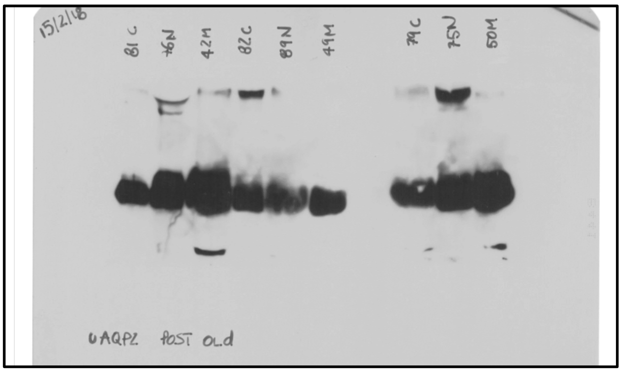

5/10/17 young
15/02/18 Old

SUPPLEMENTARY FIG. 6

Original unmodified blot (GAPDH blue arrows) in Fig. 6 (main text).

Ctrl 6 months	NaCl 6 months	MSG 6 months	Ctrl 18 months	NaCl 18 months	MSG 18 months

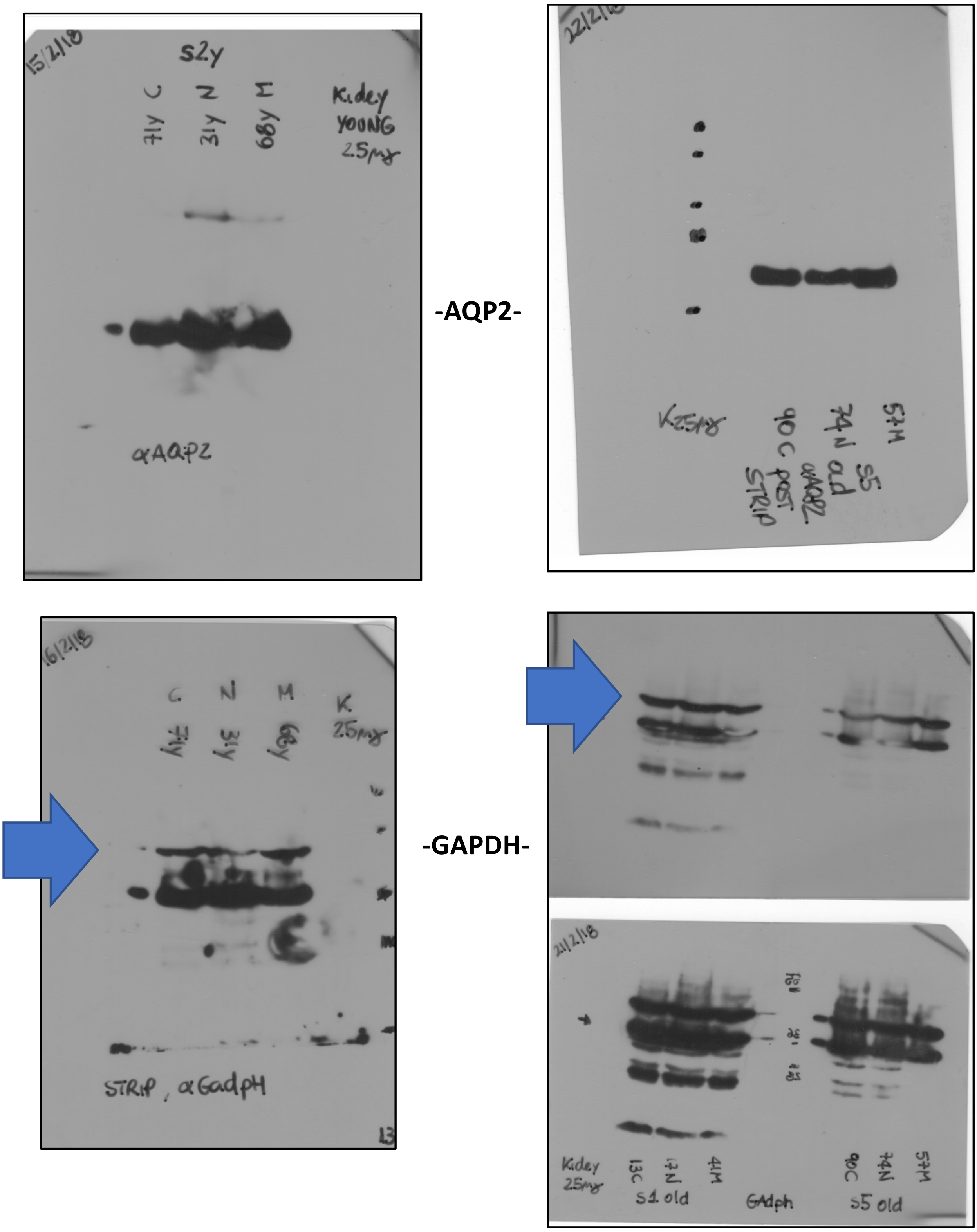

15/02/18, s2 young

21-22/02/18, s1 old

SUPPLEMENTARY FIGURE 7
Original gels for Supplementary Figure 1A

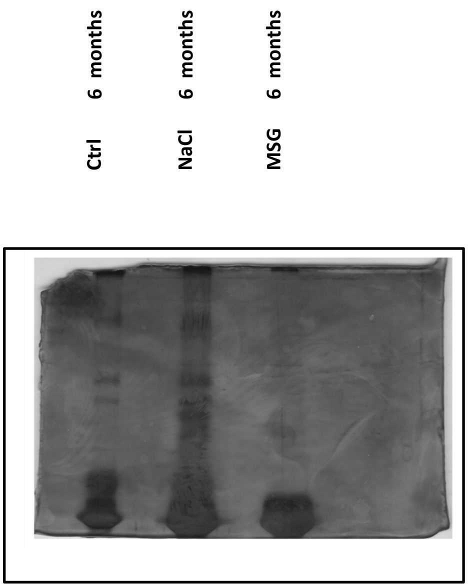

27/10/17

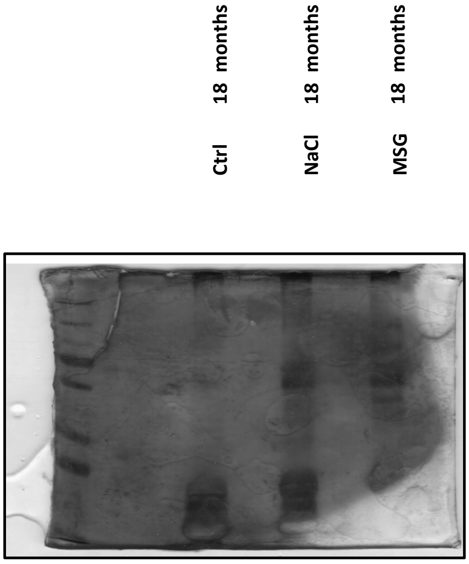

11/01/2018
